# *Halocoryza* Alluaud 1919, sea-side beetles of the Indian, Atlantic (*sensu lato*), and Pacific Oceans: a generic synopsis and description of a remarkable new species from Baja California Sur, México (Coleoptera, Carabidae, Scaritini, Clivinina)

**DOI:** 10.3897/zookeys.127.1748

**Published:** 2011-09-08

**Authors:** Terry L. Erwin

**Affiliations:** Hyper-diversity Group, Department of Entomology, MRC-187, National Museum of Natural History, Smithsonian Institution, Washington, P.O. Box 37012, DC 20013-7012, USA

**Keywords:** Sea of Cortés, Caribbean Sea, Gulf of México, intertidal zone, sandy beaches, beetles, centipedes, coral reefs, Isla Carmen

## Abstract

Information on the three previously described species of *Halocoryza* Alluaud is updated and a new species for the genus from Isla Carmen, Sea of Cortés, Baja California Sur, México is described. *Halocoryza whiteheadiana* **sp. n**. was found at UV light on a beach of that island. This species does not fit the profile of the other three species, i.e., living on coralline beach sands, or in the Mangrove intertidal zone. Two alternative possibilities as to why this is so are suggested and a study plan for testing these possibilities is proposed.

## Abstract (es)

Información de las tres especies de *Halocoryza* Alluaud previamente descritas se actualiza y una especie nueva para el género se describe de la Isla de Carmen, Mar de Cortés, Baja California Sur, **México.** *Halocoryza whiteheadiana* **sp. n.** se encontró a luz UV en la playa de esa isla. Esta especie no corresponde con los perfiles de las otras tres especies (por ejemplo, viviendo en arenas coralinas de playa o en la zona intermareal en el manglar. Dos posibles alternativas para la razón de esto son sugeridas y un plan de estudio para probar estas posibilidades se propone.

## Introduction

*Halocoryza* beetles belong to the subtribe Clivinina and are closely related to the genus S*chizogenius* Putzeys 1846, the so-called Rib-headed Beetles that D.R. Whitehead revised for his doctoral dissertation and treated in subsequent publications (Whitehead 1966, 1969, 1972; Whitehead and Reichardt 1977). During a review of collections for Volume 3 of my series of books on the Western Hemisphere Caraboidea (Erwin 2007; Erwin and Pearson 2008), I discovered in the National Museum of Natural History collection a remarkable new species of *Halocoryza* from Isla Carmen in the Sea of Cortés, Baja California Sur, México.

It is named here in honor of Donald R. Whitehead† who had a great interest in this group of beetles and discovered a lot about their special place on the sea shore. I have personally collected but one specimen of this genus on the shore of the Caribbean side of Panamá, and was amazed that these beetles accept such saline conditions, as they do. Whitehead pondered whether *Halocoryza* should be included within *Schizogenius*, perhaps as a subgenus, as is *Listropus* Putzeys, a group that Whitehead and Reichardt (1977) ranked as such. Unfortunately, Whitehead was not able to continue with this study due to his early death. Here, I am retaining the generic status he left in legacy because I believe ecological shift, in addition to structural and physiological attributes, should be an important element in deciding classification status. A shift to salt from fresh water is one that must be difficult. Living in a very saline habitat requires markedly specialized characteristics, both physically and physiologically (Kavanaugh and Erwin 1991). When additional specimens become available, especially males, classification of this species needs to be revisited.

## Methods and specimens

Methods and species concepts follow those previously described (Erwin and Kavanaugh 1981; Kavanaugh and Erwin 1991). The species validation and diagnosis format follows as closely as possible that suggested in Erwin and Johnson (2000). Measurements of length (ABL, SBL) and width (TW) follow those of Ball (1972) and Kavanaugh (1979): ABL (apparent body length), measured from apex of labrum to apex of longer elytron; SBL (standardized body length), equals the sum of the lengths of the head (measured from apex of clypeus to a point on midline at level of the posterior edge of compound eyes), PL (pronotal length ), measured from apical to basal margin along midline, and LE (elytron length), measured from apex of scutellum to apex of the longer elytron; and TW (total width), measured across both elytra at their widest point with suture closed.

Included in this study are a total of 12 specimens from the National Museum of Natural History, Washington, DC (NMNH) in my charge, and a single paratype from the California Academy of Sciences (CASC, David H. Kavanaugh, Curator). The habitus images of the adult beetles portray most of the character states referred to in the key provided. Illustrations of male genitalia (modified from Vinson 1956 and Whitehead 1966) are standard for descriptive taxonomy of carabid beetles. The habitus images of the adults were made with a Visionary DigitalTM high resolution imaging system. Figure captions include an ADP number, which is a unique identification number for the specimen that was illustrated or imaged and links the specimen and associated illustrations and/or image to additional information in electronic databases at the NMNH.

Geographical data are presented for species based on all known specimens available at the time of manuscript preparation, including those in the literature. Georeferences have been determined from locality information provided on specimen labels; only those exact georeferences that are provided on the label are placed in quotes, otherwise I have estimated these as closely as possible from places, mileage, etc. listed on the label and searched with Google Earth. Latitude and longitude are reported in decimal degrees. Here, English vernacular names are proposed, as common names are becoming increasingly needed in conservation and/or agricultural and forestry applications, and for the Encyclopedia of Life (www.eol.org).

## Accounts of taxa

### Halocoryza Alluaud, 1919 Saline Catarrh Beetles

#### Halocoryza

Alluaud, 1919: 100

http://species-id.net/wiki/Halocoryza

##### Type species.

*Halocoryza maindroni* Alluaud, 1919:101

##### Number of species.

Four

##### Taxonomy.

Stable. Adelphotaxon: *Schizogenius* Putzeys, 1846

##### Geographic Distribution.

Equatorial to Tropic of Cancer; sea coasts and islands of east Africa – Comoros – Mayotte; Djibouti; Madagascar; Mauritius; Saudi Arabia; Somalia; and natural invasive from the Caribbean into west Africa – Cameroon; Ecuador – Galapagos Islands; Barbados; Brazil – Pernambuco; Dominican Republic; Grenada; Guadeloupe; Jamaica; México – BJ, GO, QR, YC; Panamá; Puerto Rico; USA – FL; Virgin Islands – St. John, St. Thomas

##### Habitat.

Sea beaches and mangrove intertidal zone

##### References.

Bruneau de Miré (1979), Lorenz (2005), Peck (2006), Vinson (1956), Whitehead (1966)

##### Note.

The common name, Saline Catarrh Beetles, proposed here follows my principle of translating the scientific name as strictly as possible. In this case, “coryza” comes from the Greek, *koryza*, meaning cold, catarrh, as in disease. Why Alluaud named the genus so is not known.

##### Diagnostic Combination.

Differing in adult attributes from those of its adelphotaxon, *Schizogenius* Putzeys, 1846, by the following: Pygidium not striate or with very subtly crenulate striae; antennomere 2 pluristose. In addition, mandibles prominent, nearly straight laterally, abruptly angulate near apices; lacinia asetose on outer margin; frontal carinae nearly perfectly regular, parallel, equidistant, and equally raised; frons evenly convex; neck not pitted or punctate dorsally; eyes reduced, bordered laterally by a distinct carina; gula broad; mentum not deeply emarginate at middle, with median tooth obsolete and epilobes short; tarsi short; paramedian carinae of sternum II short, widely spaced and poorly developed; median lobe of male genitalia neither arcuate nor abruptly deflexed in apical third; fused stylus and coxite of the ovipositor with one robust seta (Whitehead, 1966, 1972).

##### Geographic Distribution.

Sea beaches, intertidal lagoons on the edges of mangroves, and island shores of the Atlantic, Indian, and Pacific Oceans, the Caribbean Sea, Sea of Cortés, and the Gulf of México.

##### Included Species.

The species list below, as well as arrangement of descriptions that follow is ordered alphabetically.

**Table:** 

*Halocoryza acapulcana*Whitehead 1966	Ecuador; México
*Halocoryza arenaria* (Darlington, 1939)	Barbados; Brazil; Dominican Republic; Grenada; Guadeloupe; Jamaica; México; Panamá; Puerto Rico; USA; Virgin Islands; Africa – Cameroon
*Halocoryza maindroni* Alluaud 1919	Comoros – Mayotte; Djibouti; Madagascar; Mauritius; Saudi Arabia; Somalia
*Halocoryza whiteheadiana* sp. n.	México

##### Key to the Species of Halocoryza Alluaud, 1919

**Table:** 

1	Pronotum without median sulcus, anterior angles acute; Indian Ocean	*Halocoryza maindroni* Alluaud, 1919
1’	Pronotum with median sulcus, anterior angles rounded; Atlantic, Caribbean, or Pacific Oceans	2
2(1’)	Form markedly elongate; pronotum without paramedian carinae at margins of sulcus, Sea of Cortés	*Halocoryza whiteheadiana* sp. n.
2’	Form moderately elongate; pronotum with paramedian carinae at margins of sulcus	3
3(2’)	Smaller species (LE: 1.20 – 1.35mm), elytra sparsely setose, interval 3 with 10 or fewer setae; color pale testaceous; Pacific Ocean	*Halocoryza acapulcana* Whitehead, 1966
3’	Larger species (LE: 1.35 – 1.45mm), elytra densely setose, interval 3 with 10 or more setae; color dark testaceous; Atlantic Ocean and Caribbean Sea	*Halocoryza arenaria* (Darlington, 1939)

## Species accounts

### Halocoryza acapulcana

Whitehead, 1966

http://species-id.net/wiki/Halocoryza_acapulcana

Figs 1 4, 7

Halocoryza acapulcana  Whitehead, 1966:222

#### Common name.

**Acapulco Saline Catarrh Beetle**

#### Geographic Distribution.

Native, New World. Ecuador – Galapagos Islands: Rábida (Jervis); México – OA.

#### Way of Life.

**Macrohabitat:** Lowlands, sea level, in the intertidal zone of beaches. **Microhabitat:** Adults are ground-dwelling on saline soils. **Dispersal abilities:** Macropterous, capable of flight; slow runners. **Seasonal occurrence:** Adults have been found in March and August. **Behavior:** Nocturnal predators, adults are attracted to lights.

**Figure:**
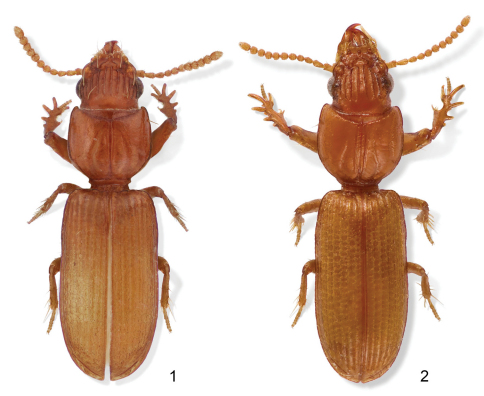
**Figure 1–2.** **1** Color image, showing habitus of *Halocoryza acapulcana* Whitehead, 1966, dorsal aspect, ABL = 2.4mm, ADP127171; Acapulco, México **2** Color image, showing habitus of *Halocoryza arenaria* (Darlington, 1939), dorsal aspect, ABL = 2.9mm, ADP116798; St. Johns, Virgin Islands.

#### References.

Peck (2006), Whitehead (1966). New data from CASC and NMNH collections.

### Halocoryza arenaria

(Darlington, 1939)

http://species-id.net/wiki/Halocoryza_arenaria

Figs 2 5, 8

Schizogenius arenaria  Darlington, 1939: 84

#### Common name.

**Sand Saline Catarrh Beetle**

#### Geographic Distribution.

Native, New World. Barbados; Brazil; Dominican Republic; Grenada; Guadeloupe; Jamaica; México – QR, YC; Panamá; Puerto Rico; USA – FL; Virgin Islands – St. John, St. Thomas; natural invasive, Africa – Cameroon.

#### Way of Life.

**Macrohabitat:** Lowlands, sea level – 1 meter altitude, on sea beaches and in the intertidal area, at or near the high tide line, and in mangrove swamps. **Microhabitat:** Adults are ground-dwelling on exposed wet substrate consisting of coquina-coral cemented by very fine silt or sand and covered with seaweed mats. **Dispersal abilities:** Wing-polymorphic: macropterous form probably capable of flight; brachypterous form, consequently flightless thus vagility limited to walking or running; both forms slow runners. **Seasonal occurrence:** Adults have been found in March – April, July, and October. **Behavior:** Adults are nocturnal predaceous halobionts and take cover in the sand or under drift and piles of seaweed on the beach. Populations of this species are associated with the centipede *Pectiniunguis halirrhytus* Crabill. In the northern part of their range, adults overwinter in the substrate; in the southern part, they likely aestivate during the dry season in the substrate.

**Figure 3.**
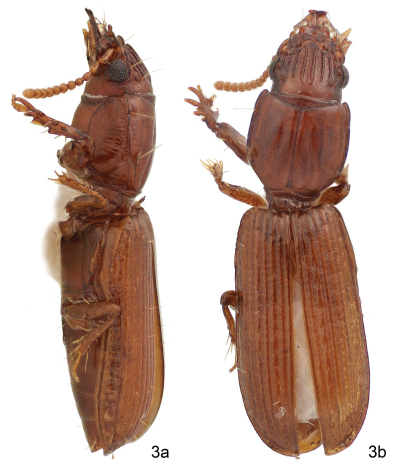
Color image, showing habitus of *Halocoryza whiteheadiana* **sp. n.**, 3a, left lateral aspect, 3b, dorsal aspect, ABL = 2.9mm; Holotype: Isla Del Carmen, BJ, México.

**Figure 4–8.**
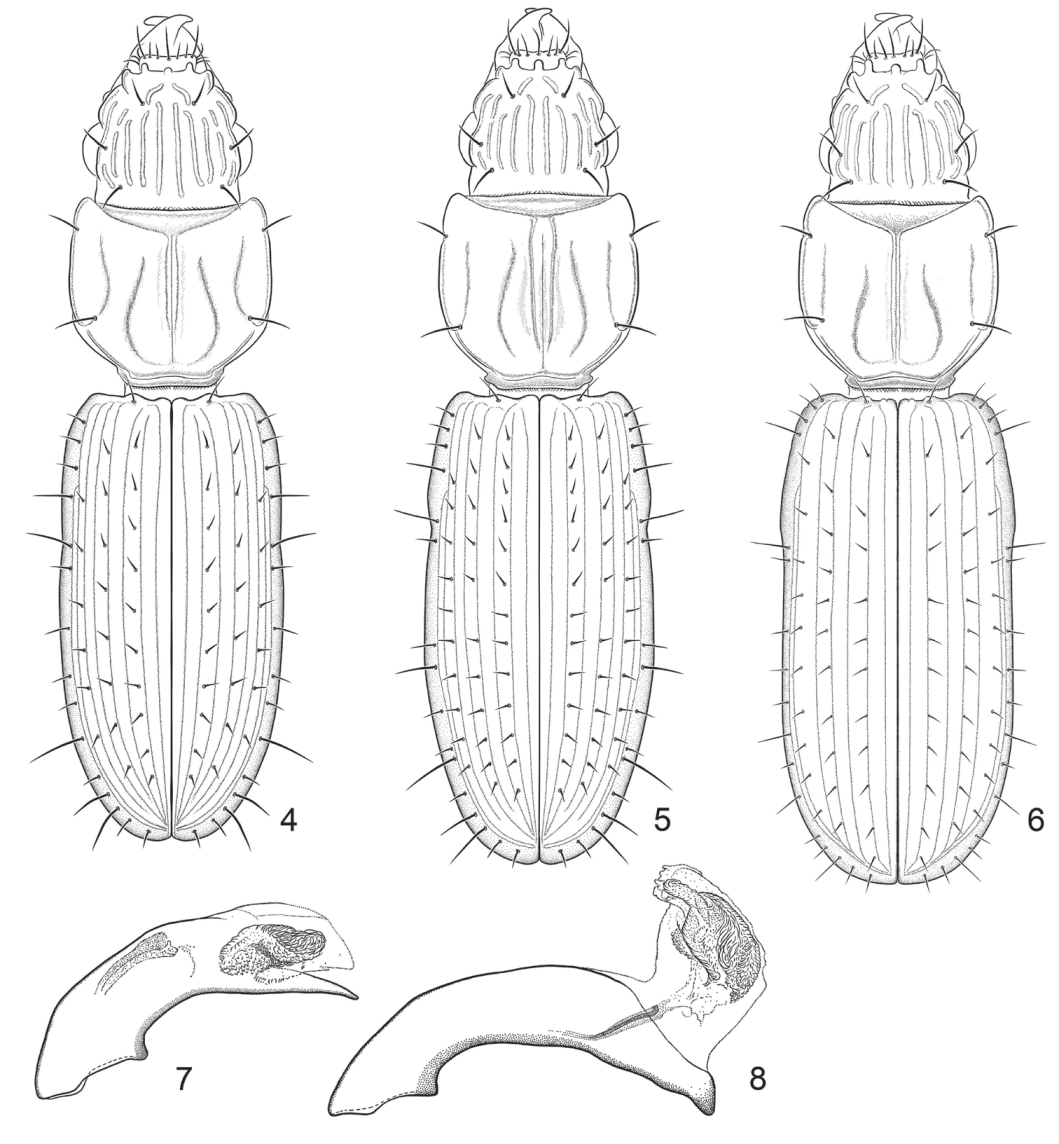
**4** Line drawing, showing habitus of *Halocoryza acapulcana* Whitehead, 1966, dorsal aspect, ABL = 2.4mm; Acapulco, México; modified from Whitehead 1966 **5** Line drawing, showing habitus of *Halocoryza arenaria* (Darlington, 1939), dorsal aspect, ABL = 2.9mm; Rio Piedras, Puerto Rico; modified from Whitehead, 1966 **6** Line drawing, showing habitus of *Halocoryza whiteheadiana* sp. n., dorsal aspect, ABL = 2.9mm; ABL = 2.8mm,ADP127139; Isla Del Carmen, México **7** Line drawing, showing male genitalia, median lobe, of *Halocoryza acapulcana* Whitehead, 1966, left lateral aspect, 0.41mm (dorsal margin of basal lobe to apex); Acapulco, México; modified from Whitehead 1966 **8** Line drawing, showing male genitalia, median lobe, *Halocoryza arenaria* (Darlington, 1939), left lateral aspect with internal sac everted, 0.41mm (dorsal margin of basal lobe to apex); Rio Piedras, Puerto Rico; modified from Whitehead, 1966.

#### References.

Bruneau de Miré (1979), Nichols (1988, Ph.D. dissertation), Peck and Thomas (1998), (Whitehead (1966, 1969).

### Halocoryza maindroni

Alluaud, 1919

http://species-id.net/wiki/Halocoryza_maindroni

Figs 9, 10

Halocoryza Maindroni Alluaud, 1919:101
Halocoryza atriceps  Alluaud, 1899:378 [not Fairmaire, 1901:5]
Halocoryza jeanelli  Vinson, 1956:313

#### Common name.

**Maindron’s Saline Catarrh Beetle**

#### Geographic Distribution.

Native, Old World. Comoros – Mayotte; Djibouti; Madagascar; Mauritius; Saudi Arabia; Somalia.

#### Way of Life.

**Macrohabitat:** Lowlands, sea level, in the intertidal zone of sea beaches. **Microhabitat:** Adults are ground-dwelling on coralline sands in the vicinity of coral reefs. **Dispersal abilities:** Wing-polymorphic: macropterous form probably capable of flight; brachypterous form, consequently flightless thus vagility limited to walking or running; both forms slow runners. **Seasonal occurrence:** Adults have been found in January and October. **Behavior:** Nocturnal predators, adults take cover during the day under dry seaweed just above the high water mark.

**Figures 9–10.**
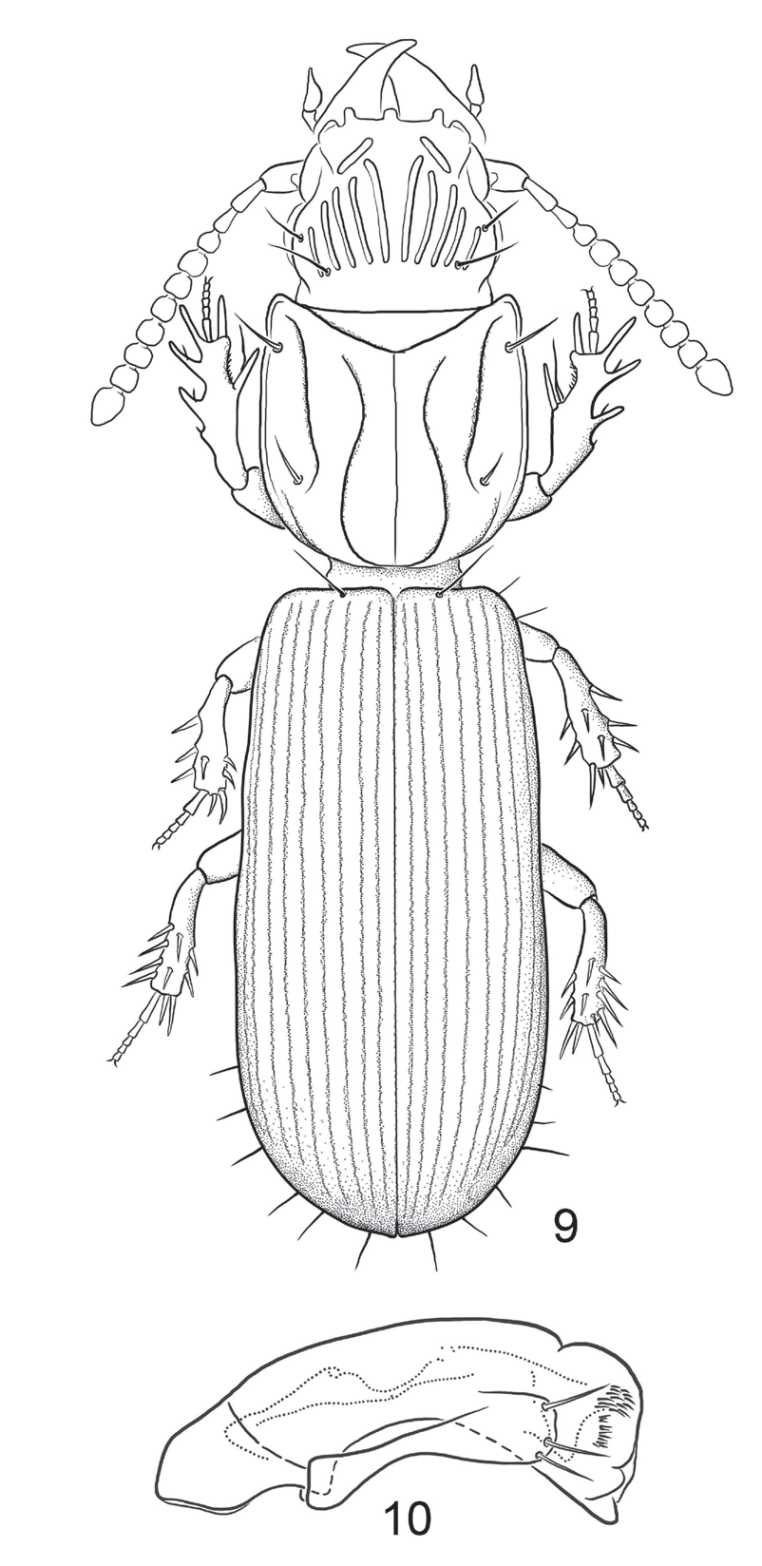
**9** Line drawing, showing habitus of *Halocoryza maindroni* Alluaud, 1919, dorsal aspect, ABL = 2.4mm; Black River, Mauritius; modified from Vinson (1956). N. B. discal setae of elytra not shown **10** Line drawing, showing male genitalia, median lobe and left paramere, *Halocoryza maindroni* Alluaud 1919, left lateral aspect, ca. 0.40mm; Black River, Mauritius; modified from Vinson (1956).

#### References.

Alluaud (1919), Jeannel (1946), Vinson (1956).

#### Note.

Vinson (1956) provided a partial description of the 3rd instar larva.

### Halocoryza whiteheadiana

Erwin sp. n.

urn:lsid:zoobank.org:act:FDB58B5D-FFB1-442F-BBEA-EBD82BC958F8

http://species-id.net/wiki/Halocoryza_whiteheadiana

Figs 3a, 3b 6 

#### Common name.

**Whitehead’s Saline Catarrh Beetle**

#### Holotype. México.

Baja California Sur, Isla Carmen, north end, sea level, approximately “26.05°N, 111.1°W,” 18-19 July 1984 (S.E. Miller) (NMNH: ADP127139, female).

#### Derivation of specific epithet.

The epithet “*whiteheadiana*” is an eponym, based on the family name of Donald R. Whitehead†, who had a profound interest in the species of this genus and its adelphotaxon, *Schizogenius* Putzeys, during his relatively short career.

#### Proposed English vernacular name.

Whitehead’s Saline Catarrh Beetle.

#### Diagnosis.

With the attributes of the genus described by Whitehead (1966) and large sized for the genus. Adults rufotestaceous and shiny throughout; shallow microsculpture only in sulci. Occiput five-carinate each side with one medial and two lateral carinae on frons. Clypeus with 3 prominent tubercles; lateral margins prominently lobate. Elytron with 10 setae in interval 3, close to interneur 2.

#### Description.

(Fig. 3). *Size*: Very small, ABL = 2.8 mm, SBL = 2.52 mm, EW = 0.69 mm, LP = 0.647mm, WP = 0.622mm, LE = 1.476mm. *Color*: Rufotestaceous throughout. *Luster*:Shiny throughout. *Head:* Labrum slightly emarginate apically, six-setose. Frons markedly tri-tuberculate apically, laterally markedly lobate, lobes nearly vertical, bicarinate basally, carinae set on an angle, widest basally. Eyes slightly convex; gena short and flat. Occiput five-carinate each side; rim above eye also carinate. *Prothorax:* Markedly convex, moderately longer than broad (W/L: 0.96), narrowed basally anteriad of posterior lateral pore; surface smooth, five-sulcate; lateral sulci 2/3 length of pronotum, extended to posterior lateral pore, paramedian sulci sigmoid shaped, not reaching base, median sulcus deep, extended from near apex to base of pronotum, slightly crossing anterior transverse sulcus; anterior and posterior lateral setae present. *Pterothorax:* Elytra markedly convex (W/L: 0.46), intervals markedly convex, intervals 3, 5, and 7 each with a serial row of setiferous punctures, 10 such in interval 3. *Legs:* Normal in female. *Abdomen*: Abdominal sterna III to VI of female with normal ambulatory setae, VII with a pair of setae each side. *Male genitalia:* Unknown. *Female genitalia*: Not studied.

#### Dispersal potential.

These beetles, as represented by the holotype, are macropterous and are capable of flight; they are slow runners, strong burrowers. However, both the Caribbean and Indian Ocean species are wing-polymorphic and perhaps with additional specimens, *Halocoryza whiteheadiana* may prove to be the same.

#### Way of life.

Adults of other *Halocoryza* species are found on coralline sands in the intertidal zone of open beaches and among mangroves; a larva of the Indian Ocean species of this genus was found under dry seaweed just above the high-water mark on coralline sands. The single known specimen of *Halocoryza whiteheadiana* was collected at UV light on a sandy beach on the north shore of Isla Carmen, Baja California Sur. Adults of *Halocoryza whiteheadiana* are likely nocturnal halobiont predators, as are members of the other known species of this genus.

#### Other specimens examined.

None.

## Evolutionary aspects

According to Whitehead (1966) and Vinson (1956) these beetles are strictly associated with coralline sands on open sea beaches, or in the intertidal zone of mangroves in the Indian, Atlantic, and Pacific Oceans, the Caribbean Sea, and the Gulf of Mexico. The recently discovered exception is *Halocoryza whiteheadiana* sp. n., described herein. It was found on the north shore of Isla Carmen in the Sea of Cortés. The nearest living reef to its type (and only known) locality is 350 km to the south at Bahia Pulmo. According to Markes E. Johnson (pers. comm.) the present sandy beaches on the north side of Isla Carmen “are exclusively carbonate sands derived from crushed mollusk shells.” Therefore, the question arises: Has *Halocoryza whiteheadiana* undergone an ecological shift from coralline sands to crushed mollusk shell sands by way of evolving its more cylindrical and elongate form. Alternatively, is it a remnant species left over after more extensive corals that prehistorically occupied the more northern part of the Sea of Cortés became extinct? According to Johnson (pers. comm.), “fossil corals are to be found on Isla Carmen, and during the latest Pleistocene, ”reefs”, formed by *Porities panamensis* (Verrill) did develop at several localities on that island in Balandra Bay, Marquer Bay, and along the south end of the island. Pliocene corals are common, but I would not say they formed reefs …” Many south-facing beaches are composed of rhodolithic sand (Johnson and Ledesma-Vázquez 2009). While all of these types of beaches are derived from different animals (corals and mollusks), or red algae (rhodolithic), they all are fundamentally calcium carbonate. For *Halocoryza* species, it may be a case not of calcium carbonate, but rather of texture that is important, i.e. grain size and shape. Exploration of beaches on other islands and those near Bahia Pulmo are likely to produce more specimens (perhaps additional species); careful analysis of the sea-side substrate will be important to test the alternate suppositions made above, i.e. remnant species, or adaptive species. In addition, more specimens will test the hypothesis that this new species, like two others in the genus, is wing-polymorphic.

Thanks to information provided by my good friend and colleague, Rick Brusca, and his colleagues Markes E. Johnson and Ramon Andres Lopez Perez, I now know that Isla Carmen has fossil coral deposits on it and that corals in the past were more extensive in the Sea of Cortés. Today, they occur in the waters off Isla Carmen, but do not form reefs there. Thus, species of the genus *Halocoryza* could be indicators of both present and/or past corals in the adjacent seas that are presently contributing, or have in the past, to the sandy mix of the beach on which they are found. Alternatively, *Halocoryza whiteheadiana* may represent a species that has undergone an ecological shift since the Pleistocene to an existence on another form of calcium carbonate, namely crushed mollusk shells. Determining if they also occur on the rhodolithic sands, (i.e. those of derived from coralline alga which are major contributors of CaCO3 to beaches in the area) will require an additional sampling.

## Supplementary Material

XML Treatment for Halocoryza

XML Treatment for Halocoryza acapulcana

XML Treatment for Halocoryza arenaria

XML Treatment for Halocoryza maindroni

XML Treatment for Halocoryza whiteheadiana
